# Evaluating the performance of surfactant and charcoal-based cleaning products to effectively remove PAHs from firefighter gear

**DOI:** 10.3389/fmats.2023.1142777

**Published:** 2023-05-11

**Authors:** MD Tanjim Hossain, Arjunsing G. Girase, R. Bryan Ormond

**Affiliations:** Textile Protection and Comfort Center (TPACC), Wilson College of Textiles, North Carolina State University, Raleigh, NC, United States

**Keywords:** PAHs, detergent, surfactant, charcoal, decontamination, firefighters, turnout gear, aromatic hydrocarbons

## Abstract

Firefighters regularly respond to fire scenes where a mixture of chemicals including volatile, semi-volatile, and nonvolatile compounds are present in smoke and soot. Polycyclic aromatic hydrocarbons (PAHs) are common contaminants at fire scenes that may be deposited on the gear and the individual firefighter. Laundering is a common approach for the decontamination of contaminated gear. Surfactants are widely used by firefighters during laundering to remove PAHs as they are generally non-toxic and biodegradable. The removal of PAHs depends on the surfactant types, chemistries, and concentrations. This study evaluated the effect of surfactant concentrations to remove persistent contaminants like PAHs from turnout gear. The cleaning performance of different types of surfactants was also evaluated. Outer shell fabrics were contaminated with a standard mixture of 16 PAH compounds, and two commercial detergents were used at different concentrations. Additionally, the cleaning efficacy of eight commercially available regular and charcoal-based cleaning products was also determined against PAHs at a single surfactant concentration. For the decontamination method, a bench-scale washing procedure simulating the National Fire Protection Assocation 1851 laundering process was used. The removal efficacy of high molecular weight (HMW) PAHs were found to be lower compared to the low molecular weight PAHs for any type or any concentration of detergent. Our research also showed that the recommended surfactant concentrations provided by detergent manufacturers can be ineffective at removing the HMW PAHs from heavily contaminated fabric. With 1mL of detergent in a 100-mL bath, which is multiple times higher than recommended amount, only 40% of HMW PAHs were removed. The cleaning efficacy can be increased to above 90% by using higher concentrations of detergents. This research shows that firefighters may need to use a higher concentration of detergent than the recommended amount to effectively remove PAHs from the gear. All the regular and charcoal-based detergents were able to remove PAHs effectively from contaminated fabrics when a higher concentration of detergent was used.

## 1 Introduction

Firefighting is one of the most hazardous occupations in the world. The chance of death, fatal injuries, skin burns, and heat stress-related casualties is common among firefighters (Mandal et al., 2022; Mandal et al., 2021; Mazumder et al., 2022). Apart from heat-related injuries, firefighting is also associated with several health hazards for firefighters owing to chemical exposure. The International Agency for Research on Cancer (IARC), an agency of the World Health Organization (WHO), reclassified the firefighting occupation as “carcinogenic to humans” (Group 1) in 2022 due to the carcinogenicity involved in occupational exposure as a firefighter during and after firefighting activities (Demers et al., 2022). Previous research showed that the chance of developing prostate cancer and mesothelioma are double among firefighters compared to general population (LeMasters et al., 2006; Kang et al., 2008; Daniels et al., 2014; Tsai et al., 2015). An increased risk of lung, bladder, and colorectal cancers is also observed among firefighters (Daniels et al., 2015; Jalilian et al., 2019; Soteriades et al., 2019; Casjens et al., 2020). Besides cancers, firefighters also suffer from respiratory diseases, reproductive system problems, skeletal, and lymphatic diseases (LeMasters et al., 2006; Daniels et al., 2015; 2014). The U.S. Forest Service reported a higher possibility of death associated with cardiovascular disease and a higher risk of lung cancer among firefighters for long exposure duration in fire scenes.

Firefighters are exposed to a wide range of chemicals and particulates while performing their tasks. Chronic exposure to these contaminants is linked to this increased risk of cancer and other health complexities (Fent and Evans, 2011; Kirk et al., 2011; Tsai et al., 2015; Mazumder et al., 2023). PAHs, phenols, phthalates, benzene, heavy metals, etc. are common carcinogenic substances that might be present in the smoke (Heus, 2015). PAHs are potential carcinogens that are generated from the incomplete combustion of organic fuels or substances like coal, wood, oil, and gas, among others. The United States Environmental Protection Agency (EPA) classified 16 PAHs as known, possibly, or probably carcinogenic for human health (EPA, 1982; Zheng et al., 2018). Exposure to these PAHs may have severe health impacts on firefighters. PAH exposure may cause acute toxicity including leukemia in humans (Akash et al., 2022). PAHs mixtures can link to cell damage and biochemical disruptions associated with cancer and other chronic diseases. Inhalation of PAHs may have the most severe impact on the wellbeing of human as respiratory exposure to PAHs may cause lung cancer (Kim et al., 2013). Gastrointestinal and bladder cancers can be developed in human beings due to the long-term exposure to PAHs. Some PAHs become geno-toxic after metabolized to the diol epoxides which are associated with carcinogenicity and toxicity process (Lewtas, 2007; Gamboa et al., 2008). Long-term exposure to PAHs may reduce immune function, hamper kidney and liver function, respiratory problems, skin, etc. (Abdel-Shafy and Mansour, 2016). A high level of exposure to PAHs mixture may cause different short-term health conditions including eye irritation, skin irritation, diarrhea, and nausea (Unwin et al., 2006).

Among the EPA classified 16 PAHs, many PAHs are identified on the turnout gear of firefighters after participating at the fire scene (Kirk and Logan, 2015; Mayer et al., 2020). Contaminated ensembles act as a source of exposure to contaminants for firefighters as the toxic compounds generated by combustion during a fire deposit on the outer surface of ensembles. Kirk et al. measured the concentration of PAHs from turnout gear of instructors who participated in structural live-fire training sessions (Kirk and Logan, 2015). They found that the concentration of PAHs outside the turnout gear was 69–290 ng/cm^2^ (Kirk and Logan, 2015). PAHs are common contaminants in the wildland fire scene also. Cherry et al. (2021) measured an increased concentration of PAHs using skin wipes from hands and neck of wildland firefighters after attending the fire site. Dermal absorption of these contaminants may occur if these transfer and deposit on the skin during the removal of gear (Sousa et al., 2022). Two types of cleanings are recommended by the National Fire Protection Assocation (NFPA) to decontaminate ensembles: routine cleaning and advanced cleaning. On-scene gross decontamination, also referred to as preliminary exposure reduction, is a part of routine cleaning that is performed without taking off the turnout gear before returning to the fire station. On-site decontamination is performed to remove contaminants from the surface of the gear without compromising the functional ability of gear (Calvillo et al., 2019). Fent et al. (2017) used soap and water along with a scrub brush to perform on-scene decontamination. They scrubbed the turnout gear using water and dish soap which reduce the PAH level by 85%. They also found 24% and 0.5% efficiency against PAH contaminants when using dry brush decontamination and air-based decontamination methods, respectively (Fent et al., 2017). Calvillo et al. (2019) evaluated the performance of the water-only decontamination method against PAHs. The authors concluded that the water-only method is ineffective in removing PAHs. Advanced cleaning means hands or machine cleaning by applying cleaning agents. NFPA recommended performing advanced cleaning such as laundering twice a year, once every 6 months, or in case any major issues are observed with the gear after performing routine inspections.

The use of detergent has been proven as an effective technique to remove PAHs from contaminated soil by increasing the solubility of the hydrophobic organic compounds (HOCs) (Paria and Yuet, 2006; Laha et al., 2009; Peng et al., 2011). Removal of hydrophobic organic compounds like PAHs depends on the desorption of contaminants from the fabric surface as the contaminants need to be incorporated into the bulk aqueous phase when washed with water (Edwards et al., 1991). When surfactants are used to remove hydrophobic compounds from contaminated fabrics, these compounds are portioned into hydrophobic cores of surfactant micelles (Peng et al., 2011). Also, enzymes present in the surfactant improve the reactivity of the fibers besides increasing the efficacy of surfactants (Olsen and Falholt, 1998; Parajuli et al., 2021). Different detergents are used by independent service providers (ISP) and fire departments to remove contaminants generated at fire scenes. Mild detergent, dish soap, and regular laundry detergent are used in the industry to perform laundering or on-scene decontamination. Some commercially available detergents in the market are specially formulated for the fire service application such as Citrosqueeze^®^ (Solutions Safety), Doff ‘n DECON^™^ (Intelagard), and Turnout Gear Wash (Gear Wash). Some charcoal-based soaps and body washes including Sootsoap^™^ and Responder Wash (Responder Wipes) are also being formulated for use in the fire service for decontaminating skin and hair to take advantage of charcoal’s ability to adsorb chemicals. The chemistry of the cleaning products plays an important role in the cleaning performance. Stolpman et al. (2021) compared the cleaning efficacy of two cleaning solutions, Decon7 and a standard detergent to remove heavy metals such as arsenic (As), cadmium (Cd), chromium (Cr), lead (Pb), and antimony (Sb) while performing laundering. They used the manufacturers’ recommended amount for both surfactants and found that Decon7 performed better to reduce metal content compared to the standard laundry detergent.

NFPA 1851: *Standard on Selection, Care, and Maintenance of Protective Ensembles for Structural Fire Fighting and Proximity Fire Fighting* does not provide any instruction regarding the amount of detergent that needs to be used during the laundry or on-scene decontamination process. Many detergent manufacturers also do not provide any guidelines in this regard. In that case, firefighters and fire services need to depend on their own judgment to decide how much detergent needs to be used. Although some manufacturers provide instructions regarding the amount of detergent that needs to be used during laundering, very few research studies have been conducted to determine how effectively their recommended amount of detergent can remove PAHs from turnout gear. Banks et al. (2021) assessed the effect of laundering on semi-volatile organic compounds: PAHs, polybrominated diphenyl ethers (PBDEs), and organophosphate flame retardants (OPFRs). The concentration of PBDEs and OPFRs was found almost similar before and after laundering. They also observed very small successes to remove some PAH compounds from contaminated gear and concluded that current laundering systems are not effective enough against semi-volatile organic compounds (SVOCs). Similarly, Girase et al. (2022) found very low removal efficiency against PAH compounds after performing laundry. Another concern is that firefighters follow the manufacturer-recommended concentration of detergent without considering the concentration of contaminants deposited on gear. Most surfactant manufacturers recommend using surfactants according to load size during the laundry process. However, previous research already showed that maximum surfactant sorption capacity is constant and does not depend on the soil/water weight-to-volume ratio (Liu et al., 1992; Zheng and Obbard, 2002). The sorption of surfactant on soil largely depends on surfactant concentration (Adeel and Luthy, 1995). A higher concentration of surfactant might be required against highly contaminated soil with persistent compounds like PAHs. The concentration of contaminants that deposit on the turnout gear is hard to predict and contamination levels could be different on the turnout gear of firefighters depending on the work assignment at the fire scene. Fent et al. (2017) observed the highest PAH contamination on the skin of firefighters who were responsible for the search and attack during fire extinguishment. PAHs are hard to remove from fabric due to low water solubility, and the higher concentration of these contaminants may make it more difficult to remove. In that case, the effectiveness of recommended concentration of detergent may reduce further. Therefore, a thorough investigation is required to determine whether the recommended concentration of detergent is effective enough to remove PAHs from the gear and what approach should be followed to gain the utmost cleaning efficacy against these persistent contaminants.

This study aims to evaluate the effect of the concentrations of detergents to remove PAHs from turnout gear. This will give an idea of whether the manufacturers’ recommended amount of detergents should be enough to remove PAHs effectively. The removal percentage of PAHs is determined using only water to show the contribution of detergent in removing PAHs. The cleaning efficacy is evaluated of some standard detergents (nonionic or the combination of non-ionic and anionic surfactants) and charcoal-based products (soaps and body wash) which could be used by firefighters to clean turnout gear and/or contaminated skin. The study used 16 different PAHs to understand how different chemical structures of PAHs act as contaminants. To our best knowledge, this is the first paper specifically exploring the performance of a wide range of detergents, including those with a charcoal component, regarding the impact of concentration on the ability to remove PAHs from firefighters’ gear. This will help in gaining a comprehensive understanding of the removal of PAHs which may help in modifying the approach to the removal of contaminants.

## 2 Materials and methods

### 2.1 Materials

#### 2.1.1 Fabric

For this study, a flame-resistant fabric commonly used in wildland firefighting ensembles was selected as the contamination substrate for all cleaning experiments. The Sigma^™^ fabric (Safety Components) was comprised of 5% meta-aramid/17% polyamide/6% para-aramid/32% Lenzing^®^ FR and had a silicone-based water-repellent finish. This material is similar to many structural firefighter outer shell materials and is certified to NFPA 1977, 1975, and 1951 standards.

#### 2.1.2 Chemical contaminants

A QTM PAH Standard Mix containing 16 PAH compounds shown in Table 1 was already prepared in methylene chloride. This standard mix was purchased from Sigma-Aldrich Inc. (CRM47930). The concentration of each PAH compound in the stock solution was 2,000 ng/μL. The standard mix was packaged in 2-mL amber vials and kept in the refrigerator at 4°C. Dilutions of the standard mix were made for calibration of analytical instrumentation which is explained in 2.2.4.

#### 2.1.3 Detergents

To assess the impact of detergent concentration, two commercially available detergents (CD-1 and CD-2) were used at different concentrations to decontaminate the fabric. CD-1 contains a nonionic surfactant (4-Nonylphenyl-polyethylene glycol), D-limonene, mackamide C, and glycol ether. CD-2 contains nonionic and anionic surfactants (alkyl ethoxy sulfate and alkyl sulfate, linear alkylbenzene sulfonate), amide oxide, hydrogen peroxide, and percarbonate. Although both are commercial detergents, CD-1 is developed specifically for turnout gear whereas CD-2 is a common consumer laundry detergent.

Cleaning efficacy was also determined for four regular detergents (laundry and dish soaps) (CD-1, CD-2, CD-3, CD-4) and four charcoal-based products (CD-5, CD-6, CD-7, CD-8). For that, 10 mL of each was added with 90 mL of water to perform the decontamination. CD-3 and CD-4 both contain a blend of non-ionic and anionic surfactants. The four latter products are mainly targeted for skin or hair decontamination and not necessarily for use on fabrics. However, they were included in this study to determine how soaps formulations containing charcoal compared to other surfactants. Supplementary Table S1 shows the list of cleaning products used in this experiment.

### 2.2 Method

#### 2.2.1 Bench-scale washing for decontamination

Fabric swatches (5 cm × 4 cm) were prepared from the roll of fabric. A repeater pipette (Eppendorf) was used to dispense six 5-μL droplets of the reference mix (60,000 ng of each PAH compound) on the fabric swatch. Fabric swatches were kept for 30 min in ambient conditions so that contaminants could penetrate the fabric surface and the solvent could evaporate. Contaminated swatches were then transferred into 250-mL Erlenmeyer flasks containing water, detergent, and glass beads. The combined volume of detergent and water in each flask was 100 mL. The amounts of surfactants were 0 mL (water only), 1 mL, 10 mL, 20 mL, and 50 mL which were added with 100 mL, 99 mL, 90 mL, 80 mL, and 50 mL of water, respectively. Three replicates were used for each condition. In each flask, 5 g of 4-mm glass beads were added to provide some mechanical agitation during washing. The flasks were placed in an LSE Corning^®^ bench-top shaking incubator to perform bench-scale washing of contaminated fabric swatches. All the samples were washed for 60 min at 40°C and 300 RPM which was the maximum RPM available on the shaker bath. This high RPM would provide mechanical agitation during the washing process. The temperature 40°C was used to perform washing according to the NFPA 1851 standard (NFPA, 1851). After 1 h of washing, contaminated water was drained, and samples were rinsed with 100 mL of clean water at room temperature for 10 min. In each batch, nine samples were washed using the bench-scale washer-extractor. Then, samples were placed in a rack for 24 h for air drying.

#### 2.2.2 Pressurized solvent extraction

The Buchi Speed Extractor E–916 was used to perform the extraction of each fabric. Extraction was completed for one cycle which consists of a 1-min heat up, a 5-min hold, and a 2-min discharge. Temperature and pressure were held at 100°C and 100 bar, respectively. The extraction solvent was n-hexane (95% Millipore Sigma). Fabric swatches were inserted into a 10-mL stainless steel extraction cell and the rest of the volume of the cell was filled with glass beads to reduce the consumed amount of solvent during extraction. Top and bottom cellulose filters were used to cap the 10-mL extraction cell. This will trap the particulate present in the fabric samples so that extracted solution is contamination free. One positive control (known quantity of contaminants without wash) was used during each extraction cycle to monitor and ensure that extraction cycles were working properly. It took 18 min to complete the extraction process. After the extraction, n-hexane was added to the extracted solution and then the solution was diluted to volume in a 10-mL volumetric flask. The diluted extract was then transferred into a 2-mL amber autosampler vial using a 3-mL Luer-lock syringe with 0.2 μm PTFE filters.

#### 2.2.3 Gas chromatography-mass spectrometry (GC-MS) analysis

Samples were analyzed using Agilent 7890B gas chromatographic system connected with Agilent 5977B mass spectrometer equipped with electron ionization (EI). The splitless mode was used for the chromatographic analysis with a 100 mL/min purge flow at 1 min. Samples were analyzed using an Agilent EPA 8270D fused silica capillary column (30 m × 0.25 mm × 0.25 μm). Helium was used as the carrier gas with a 1.2 mL/min flow rate. The Agilent Ultra Inert liner (5190–3136) was used in the GC inlet. The injection temperature and injection volume were 250°C and 1 μL, respectively. Initially, the oven temperature was 40°C and raised to 200°C at 10°C/min followed by a 1-min hold. Then, the temperature was again increased to 300°C at 25°C/min and held for one more minute. The total run time for each sample was 30 min. Samples were analyzed in scan mode (35–550 amu) and 70 eV ionization energy was used in EI mode. Throughout the run, the MS transfer line, MS quad, and ion source temperatures were 280°C, 300°C, and 200°C, respectively.

#### 2.2.4 Calibration curve preparation

The instrument was calibrated for each compound. Calibration solutions were prepared in six concentrations: 0.2 ng/μL, 0.8 ng/μL, 2 ng/μL, 4 ng/μL, 6 ng/μL, and 8 ng/μL. To prepare calibration solution, shown in Table 2, PAHs mixture was injected into 10-mL volumetric flasks and diluted using n-hexane. Each concentration was analyzed three times and the average was taken to minimize error. The minimum R-square coefficient was 0.997 among all the calibration curves from each compound. The lowest concentration (0.2 ng/μL) was run seven times consecutively to get the limit of detection (LOD) and limit of quantitation (LOQ) for each compound. Equations 1 and 2 were used respectively to calculate the LOD and LOQ:

(1) $$LOD=\frac{3\sigma }{\text{m}}$$

(2) $$LOQ=\frac{10\sigma }{m}$$

Here, σ = standard deviation of the peak area of seven consecutive runs and m is the slope of the calibration curve for each compound. LOD represents the minimum value at which the instrument can confidently identify instrument noise and peak area. LOQ represents the minimum value at which the instrument can quantify the peak area of a compound.

#### 2.2.5 Determination of extraction efficiency

Two fabric swatches were spiked with 60,000 ng using the same procedure described as above and were extracted. These swatches were considered as positive controls. The peak area obtained for each compound was used to obtain the concentration using the calibration curve. The spiked amount of contaminant was compared with the extracted amount of contaminant to calculate the extraction efficiency. Uncontaminated fabric washed with surfactant is considered as the negative control.

#### 2.2.6 Determination of cleaning efficacy

Cleaning efficacy represents the concentration of contaminant removed by the washing process. Unwashed contaminated fabric samples were compared to uncontaminated negative samples to calculate cleaning efficacy. Eq. 3 was used to calculate cleaning efficacy which was taken from NFPA 1851 standard after some modification.


(3) $$Cleaning\;efficacy=\frac{\left(Cc-Cm)-(Cw-Cp\right)}{\left(Cc-Cm\right)}\times 100$$

Cc = Original concentration of contaminant dosed on the fabric.

Cm = Amount of contaminant on the unwashed uncontaminated fabric.

Cw = Amount of contaminant on washed fabric.

Cp = Amount of contaminant on uncontaminated washed fabric.

## 3 Result

PAHs are classified into two groups depending on the number of rings present in the structure of the compounds: high molecular weight (HMW) PAHs and low molecular weight (LMW) PAHs. In this analysis, HMW PAHs contain four or more aromatic rings in the structure whereas LMW PAHs contain two or three aromatic rings (Lee, 2010). The concentration of each compound was considered zero in unwashed uncontaminated fabric (Cw) and uncontaminated washed fabric (Cp) if no value was obtained after GC analysis. If no chromatograms are detected in GC-MS in the washed sample, then LOQ/2 values were used to calculate cleaning efficacy. The error bars in all the graphs represent the standard errors.

### 3.1 Cleaning efficacy of only water

**FIGURE 1:**
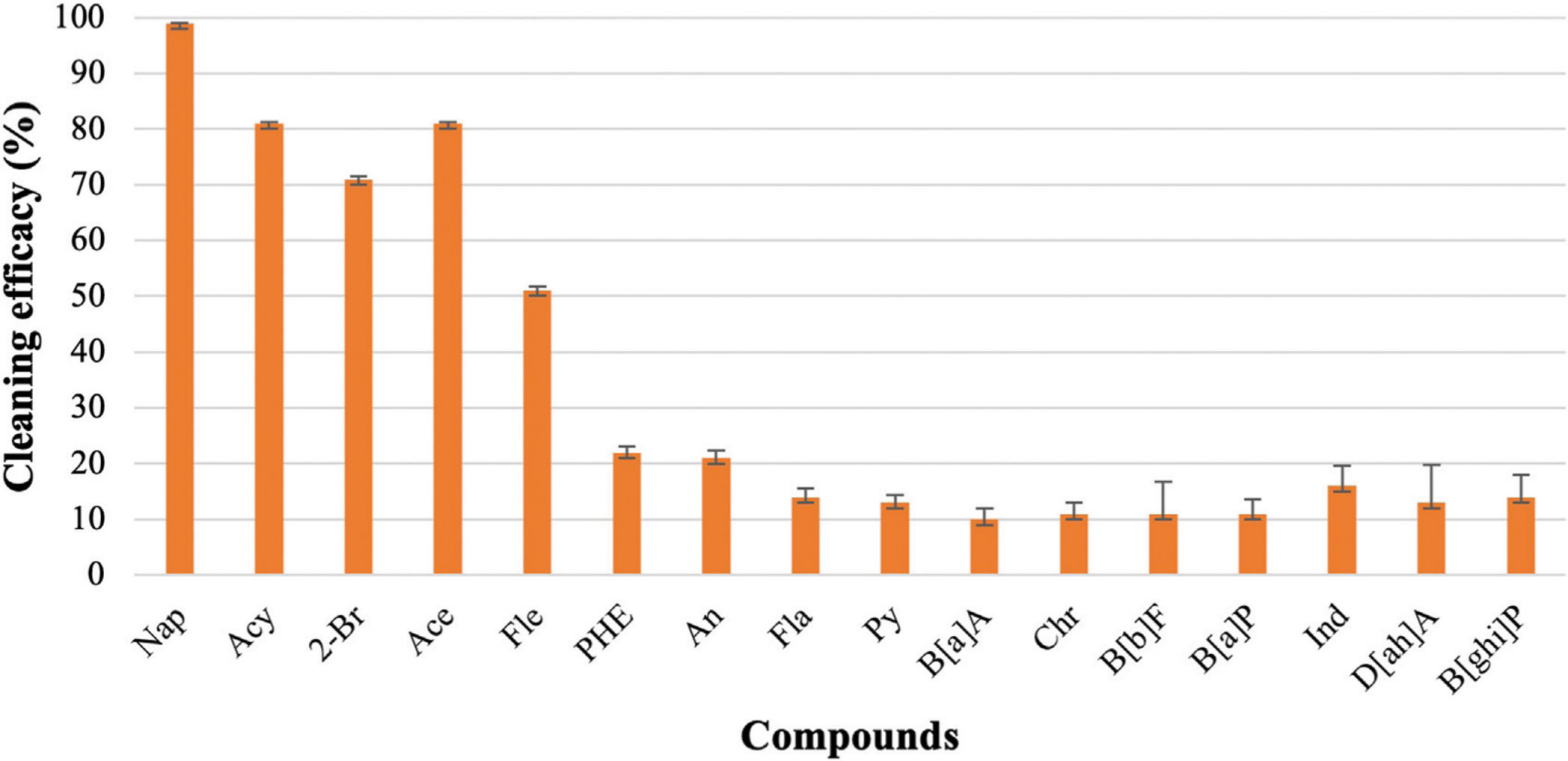
Cleaning efficacy of water against 16 PAHs [range = mean ± standard error (SE)].

Among the 16 PAHs, seven LMW PAHs are naphthalene (Nap), acenaphthylene (Acy), 2-bromo naphthalene (2-Br), acenaphthene (Ace), fluorene (Fle), phenanthrene (PHE), and anthracene (An). Other eight are HMW PAHs: fluoranthene (Fla), pyrene (Py), benz-a-anthracene B[a]A, chrysene (Chr), benzo-b-fluoranthene (B[b]F), Benzo-a-pyrene (B[a]P), Indeno-1,2,3-cd pyrene (Ind), Dibenz-a,h-anthracene (D[ah]A), and Benzo-g,h,i-perylene (B[ghi]P). Figure 1 shows the effectiveness of cleaning the fabrics with only water. Nap and 2-Br contain two rings in the structure, and the Cleaning efficacies of water were 95% and 75% against these compounds, respectively. Acy, Ace, Fle, Phe, and An contains three rings in their structures. The water-only removals for Acy, Ace, Fle were 81%, 81%, and 51%, respectively whereas approximately 20% cleaning efficacy was observed for both PHE and An. The average cleaning efficacy of ∑7 LMW PAHs was 70%. As for HMW PAHs, water-only process showed low effectiveness and the cleaning efficacy of water for Fla, Py, B[a]A, and Chr were 14%, 13%, 10%, and 11%, respectively. B[b]F, B[a]P contain five rings in their structure, and the water-only process removed 11% of both compounds. Ind, D[ah]A, and B[ghi]P contain six rings, and the cleaning efficacies of water against these compounds were 16%, 13%, and 14%, respectively. The cleaning efficacy of water decreases significantly against HMW PAHs, and the average cleaning efficacy of ∑9 HMW PAHs is 12.55%.

### 3.2 Effect of different concentrations of surfactants

**FIGURE 2:**
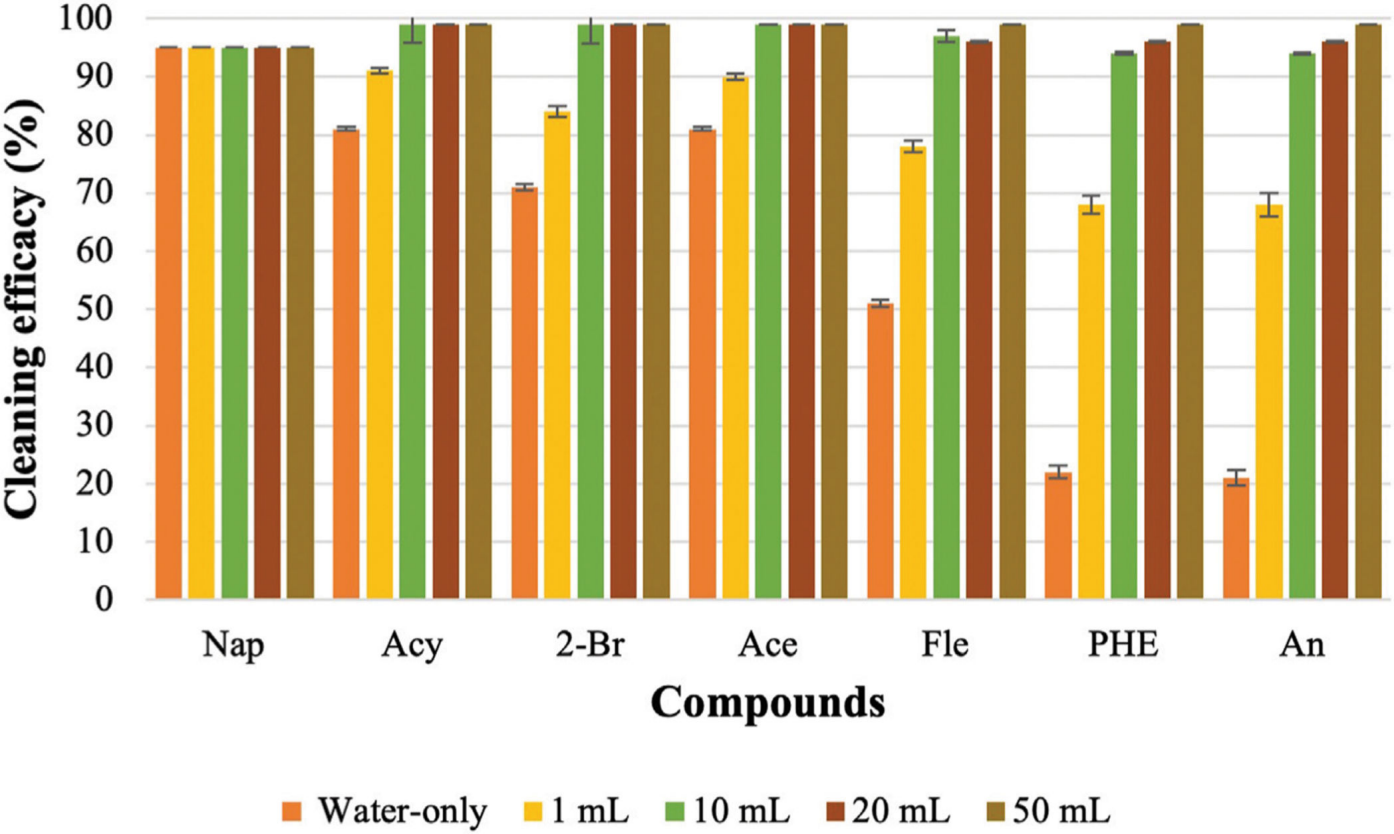
Cleaning efficacy of CD-1 against LMW PAHs [range = mean ± SE].

**FIGURE 3:**
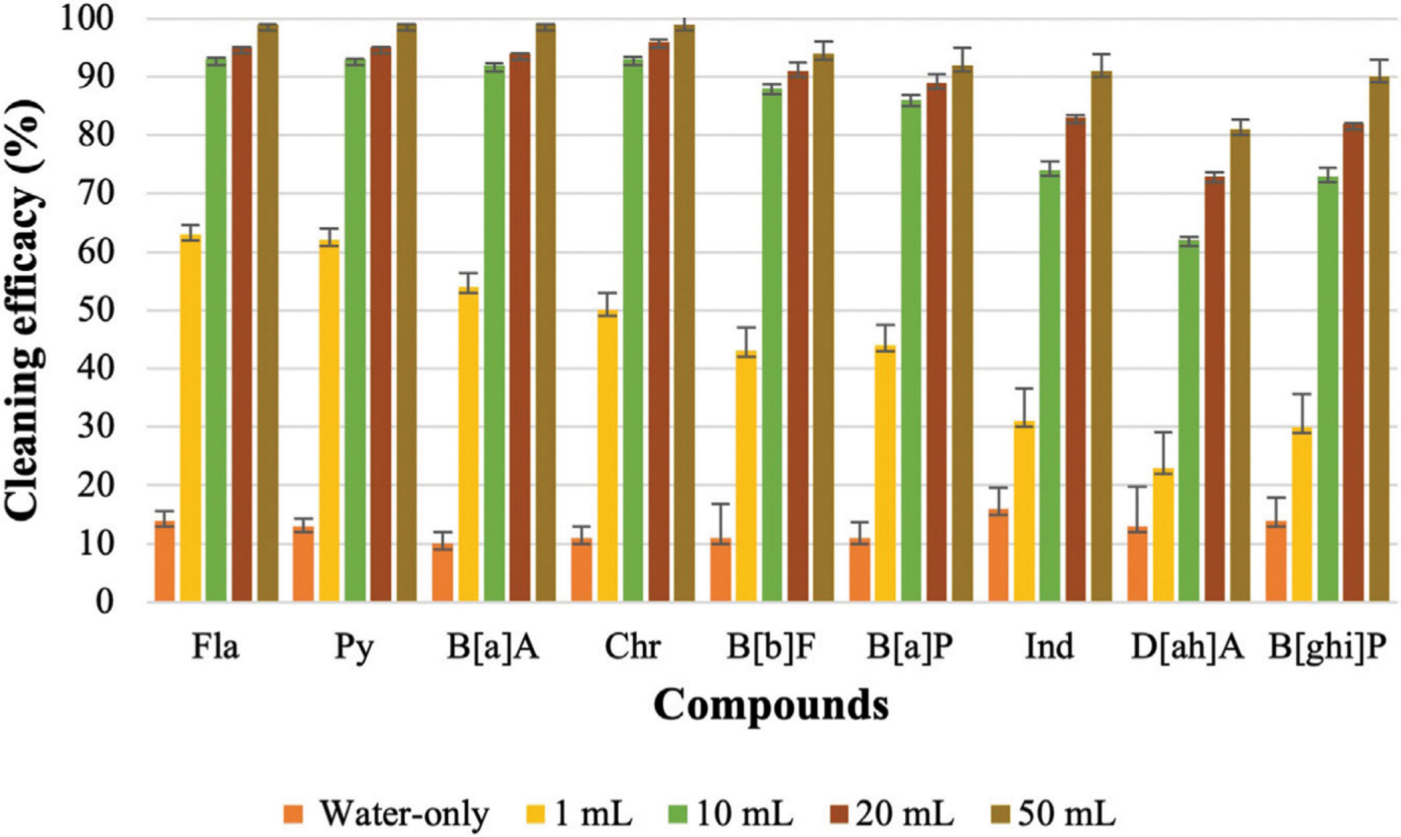
Cleaning efficacy of CD-1 against HMW PAHs [range = mean ± SE].

CD-1 is usually used to wash turnout gear and CD-2 is a popular consumer laundry detergent that is also commonly used by fire departments. According to the safety data sheet (SDS) of CD-1, 6 oz of detergent needs to be used for a 45-lbs washing load. The weight of each fabric swatch was 0.5 g. By scaling to the recommended amount, 10 μL of CD-1 needs to be added during the bench-scale washing. However, 1 mL of detergent was used as the lowest concentration of CD-1 for this experiment which is 100 times higher than the recommended amount. CD-2 does not have any specific recommendations like CD-1. Therefore, both detergents were used at the same ratios for the washing. Figure 2 and Figure 3 show the cleaning efficacy of different concentrations of CD-1 against LMW PAHs and HMW PAHs respectively.

Figure 2 shows that cleaning efficacy increased significantly for LMW PAHs with 1 mL of surfactant was used compared to the water-only process. Among the LMW PAHs, the lowest cleaning efficacies were obtained for PHE and An (22% and 21%) using only water. Cleaning efficacy against both compounds increased to 68% when 1 mL of CD-1 was used. Cleaning efficacies of other LMW PAHs also increased significantly. With the 1 mL of CD-1, Acy (91%), 2-Br (84%), Ace (90%), and Fle (78%) were all removed at high levels. For PAHs containing four rings a significant increase in removal was also observed with the 1 mL of surfactant (increased from 10% to 14% to around 50%–60%). However, very poor cleaning performance was obtained for PAHs with five or six rings in the structure. Around 30% or less cleaning efficacy was observed for Ind, D[ah]A, and B[ghi]P which are the largest structures and contain six rings.

Increasing to 10 mL of CD-1 showed a drastic improvement in cleaning efficacy against all the PAH compounds. Above 90% cleaning efficacy was measured for all the LMW PAHs and HMW PAHs containing four rings. Cleaning efficacy against all the PAH compounds increased further with 20 mL and 50 mL of CD-1. However, using even 50 mL of surfactant was not able to remove 100% of PAHs containing six rings. Using 20 mL of CD-1 removed 83%, 73%, and 82% of Ind, D[ah]A, and B[ghi]P, respectively, whereas 50 mL of CD-1 removed 91%, 81%, and 90% of those PAH compounds. With the increase of surfactant’s concentrations, the solubility of each PAH compound also increases which contributes to enhanced desorption of these PAH compounds (Edwards et al., 1991; Zhou and Zhu, 2007; Zhu and Zhou, 2008; Peng et al., 2011). This might be the reason for the increased removal of PAHs with higher concentrations of surfactants.

**FIGURE 4:**
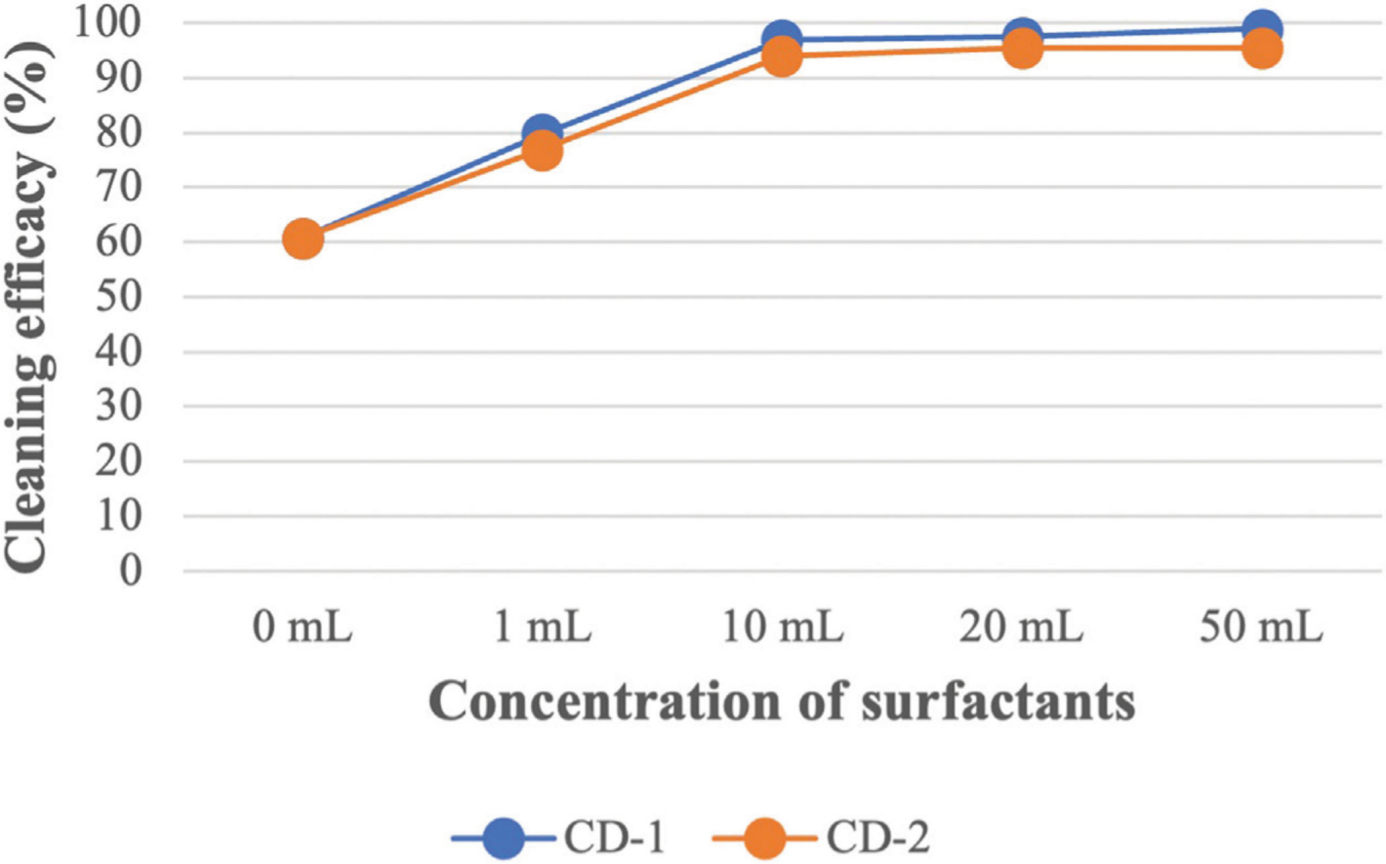
The average cleaning efficacy of ∑7 LMW PAHs using different concentrations of CD-1 and CD-2.

**FIGURE 5:**
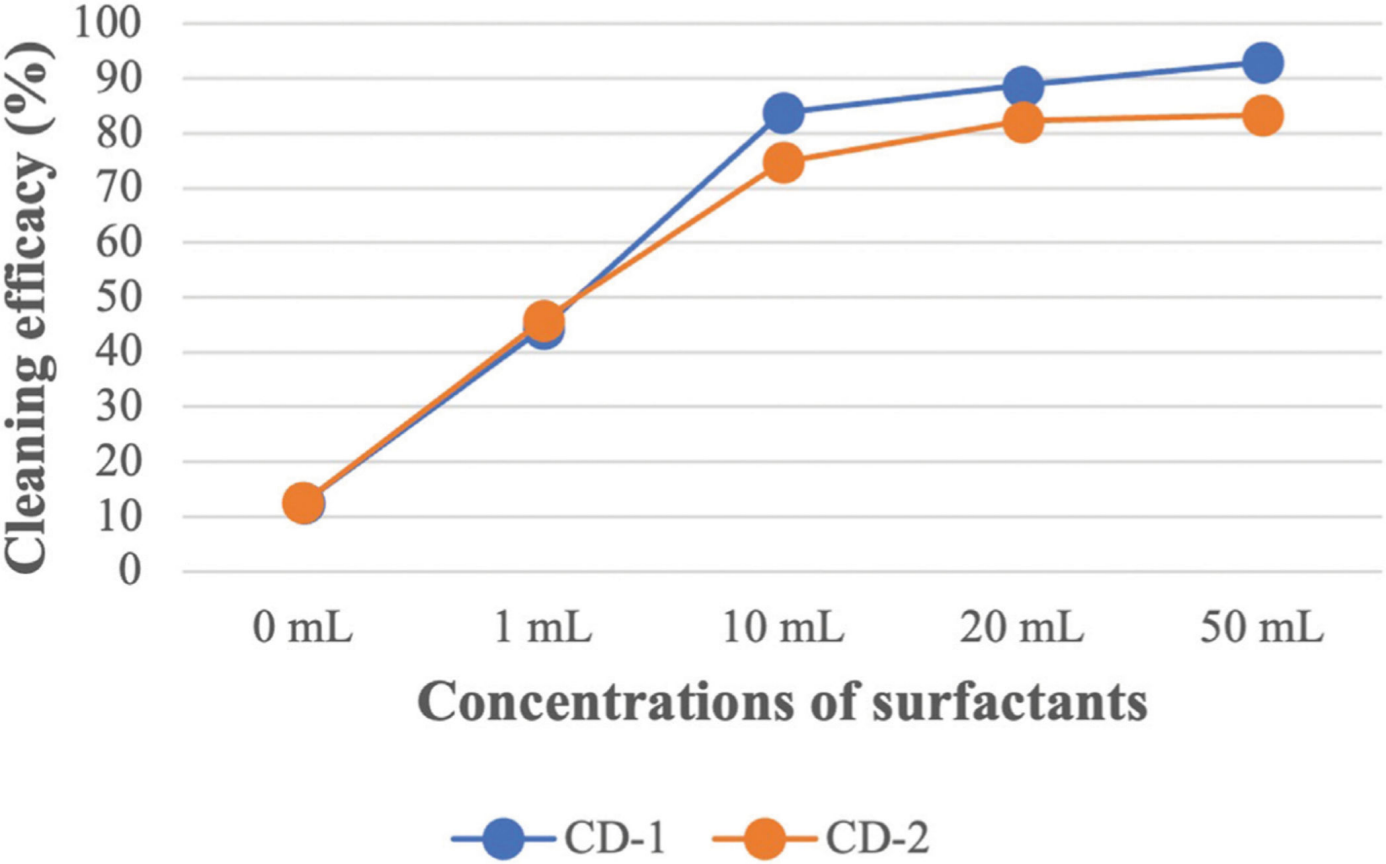
The average cleaning efficacy of ∑9 HMW PAHs using different concentrations of CD-1 and CD-2.

Like CD-1, the same concentrations of CD-2 were used against all the PAH compounds (shown in Supplementary Table S2). A similar trend in cleaning efficacy was obtained for different concentrations of CD-2. Like CD-1, 1 mL of CD-2 did not remove HMW PAHs containing five or six rings. Using 10 mL of CD-2 shows very good cleaning efficacy against all PAHs compounds although cleaning efficacy is low against D[ah]A (53%). Using 20 and 50 mL of CD-2 increased cleaning efficacy further and the highest cleaning efficacy of D[ah]A is around 69% when 50 mL of CD-2 was used. Figure 4; Figure 5 show the average cleaning efficacy of ∑7 LMW PAHs and ∑9 HMW PAHs using CD-1 and CD-2, respectively; Figure 4 shows that 83% of LMW PAHs are removed by 1 mL of CD-1 and 80% of ∑7 LMW PAHs are removed by 1 mL of CD-2. Using 10 mL or higher concentrations of CD-1 and CD-2 showed near 100% cleaning efficacy against ∑7 LMW PAHs. Using 1 mL, 10 mL, 20 mL, and 50 mL of CD-1 show 44%, 84%, 89%, and 93% cleaning efficacy against ∑9 HMW PAHs whereas 1 mL, 10 mL, 20 mL, and 50 mL of CD-2 show 46%, 75%, 82%, and 83% cleaning efficacies against ∑9 HMW PAHs (Figure 5).

### 3.3 Evaluate the cleaning efficacy of regular and charcoal-based cleaning products

**FIGURE 6:**
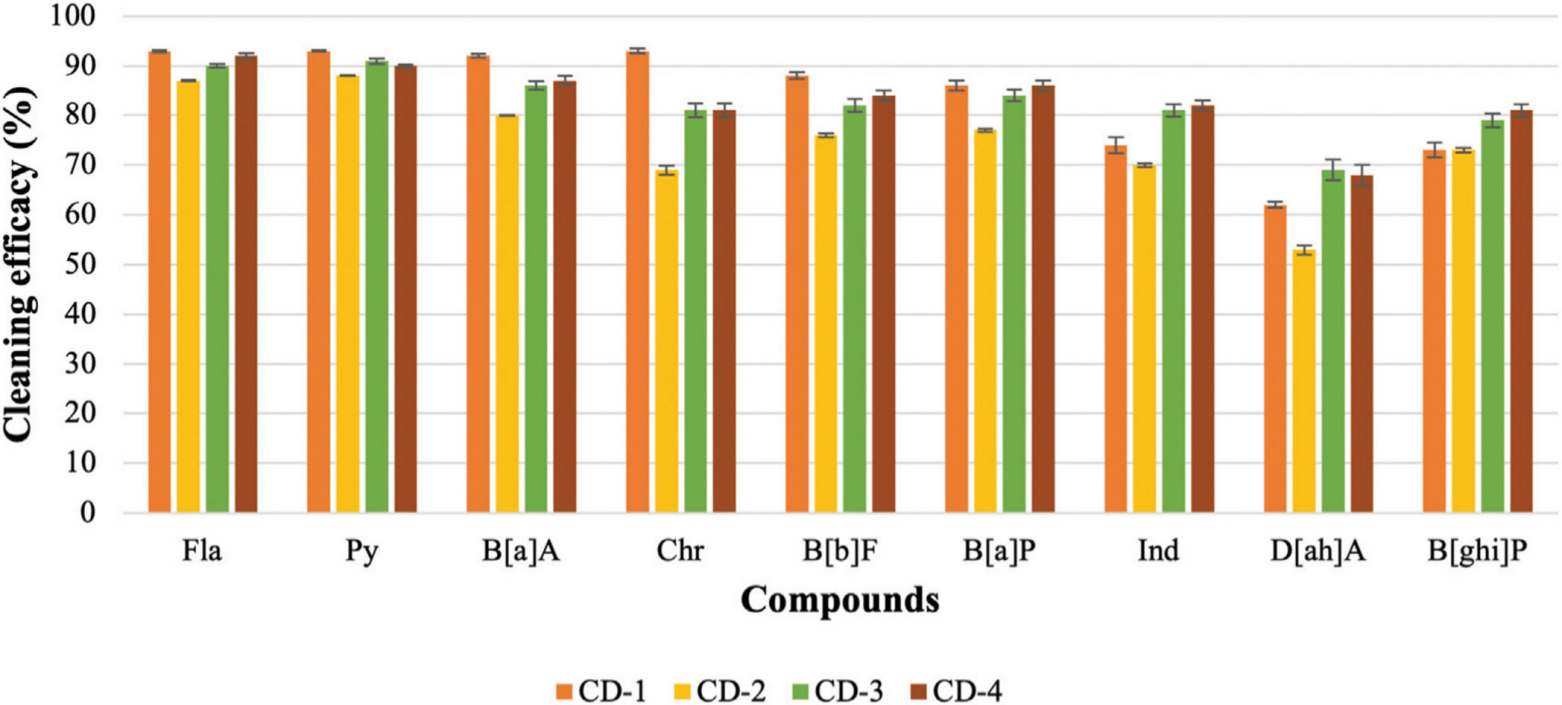
Cleaning efficacy of regular detergents against HMW PAHs [range = mean ± SE].

**FIGURE 7:**
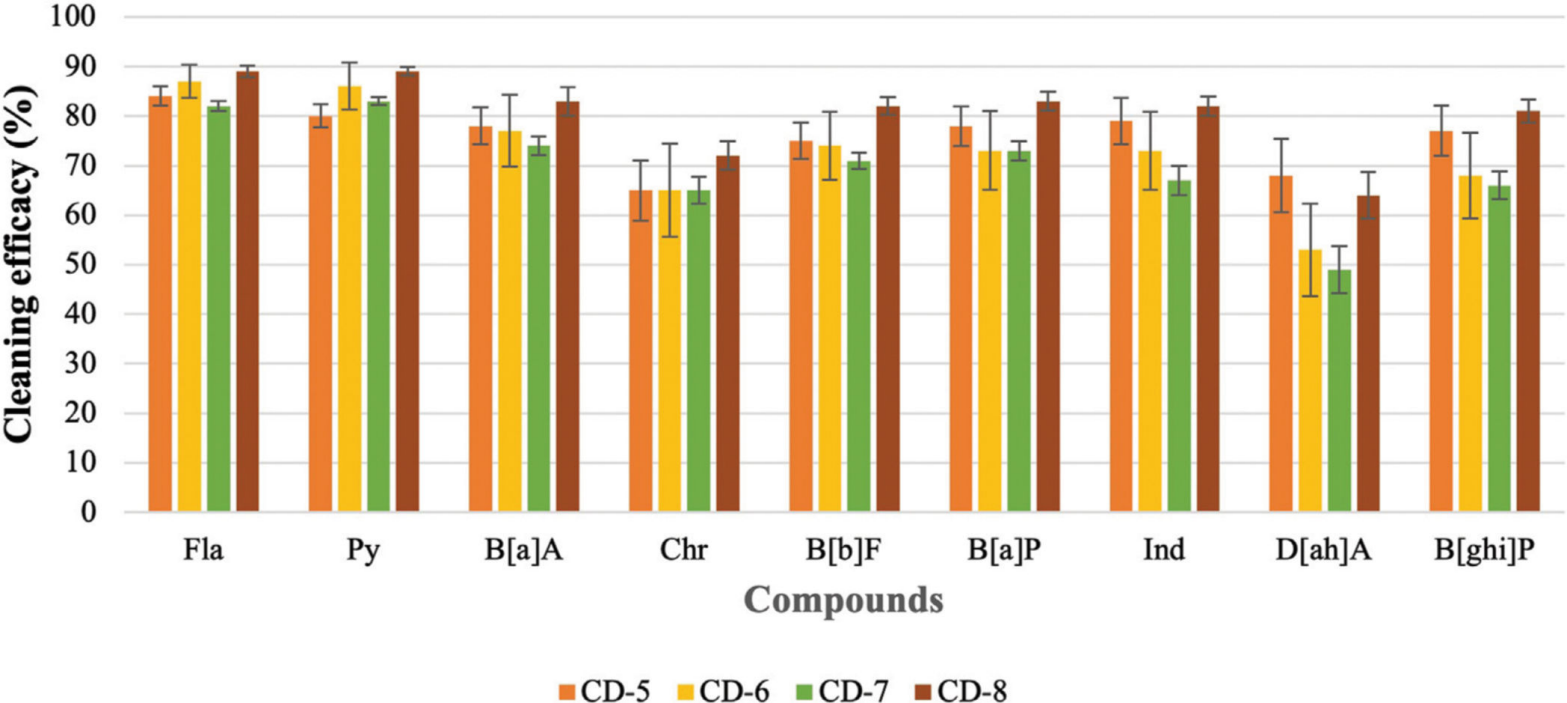
Cleaning efficacy of charcoal-based detergents against HMW PAHs [range = mean ± SE].

The previous part of the experiment shows that the cleaning efficacy of detergents increases with the increase in concentration. Therefore, it is hard to compare among detergents as one detergent may show lower or similar cleaning efficacy depending on which concentration is used. As 10 mL of detergents with 90 mL of water shows good cleaning efficacy against most of the PAHs, this concentration of detergent is used to evaluate the cleaning efficacy of regular detergent (anionic, nonionic, or cationic surfactant) and charcoal-based products. Figure 6; Figure 7 show the cleaning efficacy against HMW PAHs using regular detergents and charcoal-based products, respectively. Cleaning efficacy has been determined against all the 16 PAH compounds and removal efficacy of LMW PAHs has been shown in the Supplementary Figures S1, S2 respectively when regular and charcoal-based detergents were used. Around 90% or higher cleaning efficacy is obtained against ∑7 LMW PAHs for each regular detergent and charcoal-based product. As expected, the removal of PAH compounds decreases with the increase of molecular weight and octanol-water partition coefficient ($K_{\text{OW}}$) values. The removal of PAHs containing four aromatic rings is higher compared to those with five or six rings. The regular surfactants CD-1, CD-2, CD-3, and CD-4 show 84%, 75%, 83%, and 83% cleaning efficacy against ∑9 HMW PAHs. Regular surfactants and charcoal-based surfactants showed similar cleaning performance. The charcoal-based products CD-5, CD-6, CD-7, and CD-8 showed 76%, 72%, 70% and 81% cleaning efficacy respectively against ∑9 HMW PAHs. Charcoal-based surfactants are developed to clean body parts like hair and hands. Higher removal of PAHs from outer shell fabrics by using these products may also indicate that these products would be able to remove PAHs from the body parts of firefighters. Each detergent showed the lowest cleaning efficacy against D[ah]A which contains six aromatic rings in its structure.

## 4 Discussion

In most of the prior research works, wipes were used to collect PAHs from the outer surface of fabrics to calculate the cleaning efficacy although the efficacy of wipes to collect PAHs from ensembles was unknown. Therefore, pressurized solvent extraction of contaminated fabric was done in this research to collect contaminants from both the fabric surface and in-between the fibers. Besides that, contaminants deposit on the turnout gear unevenly at the fire scene, making it harder to compare pre and post-cleaning concentrations of contaminants. To avoid such issues, a known concentration of contaminants was pipetted on the fabric. Moreover, using the full-scale washer extractor increases the chance of cross-contamination during the washing process. Therefore, a bench scale washing method was used in this experiment to evaluate the cleaning performance. The removal of PAHs during the cleaning process largely depend on the physicochemical properties of these compounds like octanol-water partition coefficients ($K_{\text{OW}}$) and solubility. The ratio of a chemical’s concentration in the octanol phase and the aqueous phase is expressed as $K_{\text{OW}}$ which can be used to estimate the solubility of that chemical both in the aqueous and organic phases.

### 4.1 PAHs removal by water

Among the 16 targeted PAHs, seven are LMW PAHs containing two to three fused rings and the other nine PAHs are HMW PAHs containing four to six fused rings. The range of $K_{\text{OW}}$ values of LMW PAHs and HMW PAHs is 3.29–4.45 and 4.9 to 6.50. Generally, $K_{\text{OW}}$ is expressed in logarithmic form. Compounds are lipophilic or hydrophobic when $K_{\text{OW}}$ is greater than 1 and compounds are hydrophilic if $K_{\text{OW}}$ is below 1. $K_{\text{OW}}$ is inversely proportional to solubility and directly proportional to the molecular weight of any compound. Therefore, the water solubility of HWM PAHs is low compared to LMW PAHs. This is responsible for better removal efficacy of LMW PAHs compared to HMW PAHs when water or any concentrations of surfactant were used. Usually, increased temperatures during the extraction process increase the solubility of PAHs in the solvent used for extraction and accelerate the desorption of PAHs from a solid matrix (Lau et al., 2010). LMW PAHs, such as naphthalene, could be evaporated due to their higher volatile nature. Therefore, low extraction efficiency was observed among LMW PAHs compared to HMW PAHs compounds. Especially, the extraction efficiency of naphthalene (28%) was very low due to its low molecular weight, high solubility, and low boiling point. The low extraction efficiency of naphthalene was considered during the calculation of cleaning efficacy, and LOQ/2 was used to measure the concentration of naphthalene as no peaks were detected after washing with water. Considering the low extraction efficiency, the cleaning efficacy of naphthalene is around 95% with water. The cleaning efficacy of water against other LMW PAHs decreases with the increase of molecular weight and $K_{\text{OW}}$ values. However, around 61% of ∑7 LMW PAHs are removed by water only. This indicates that a significant amount of lighter and high volatile PAHs can be removed by using only water. HMW PAHs have low solubility and high $K_{\text{OW}}$ values which make them less mobile in the soil-water system compared to LMW PAHs (Sabljić et al., 1995; Lu et al., 2008). Due to the higher $K_{\text{OW}}$ values, HMW PAHs become more non-polar which indicates these compounds show more affinity toward fabrics. Therefore, the cleaning efficacy of water and other cleaning materials is low against HMW PAHs compared to LMW PAHs and the average cleaning efficacy is around 12.5% against ∑9 HMW PAHs when only water was used for the washing.

### 4.2 Effect of detergent concentration to remove PAHs

It is very important for firefighters to select appropriate detergents and concentrations to remove persistent contaminants like PAHs from turnout gear. According to the safety data sheet of CD-1, 10 μL should be used considering the weight of the fabric (0.5 g) used in the experiment. However, as the fabric is heavily contaminated, the minimum amount of CD-1 was selected as 1 mL to remove PAHs from the fabric. The same concentration of CD-2 was used as it does not have any specific guideline regarding the concentration of surfactant that needs to be used during the laundry process. To select the amount of detergents during the washing, both detergents focused on the load size without considering the concentration of contaminants in the turnout gear.

Cleaning efficacy against PAHs increased significantly using 1 mL of surfactant compared to using only water and cleaning efficacy is further increased with the increase of surfactant concentrations. Surfactants are sorbed as monomers at lower concentrations and form a monolayer on the fabric surface. Therefore, low partition of PAHs is observed from fabric surface at lower concentrations. Sorption of surfactant increases drastically when micelles are formed. The desorption of PAH compounds occurs when the concentration of surfactant is significantly higher than the critical micelle concentration (CMC) (Grasso et al., 2001). If the concentration of surfactant is lower than the CMC value, the surfactant fails to desorb PAHs from the fabric as the surfactant tends to sorb onto the fabric itself until it reaches the CMC (Grasso et al., 2001). Therefore, the solubility of most of the PAHs improves dramatically when the concentration of surfactant is above the CMC value. Surfactants are amphiphilic in nature and a higher concentration of surfactant needs to be used to form micelles in the presence of soil due to the sorption of surfactant on the fabric. The CMC of a surfactant in a soil-water system is high compared to an aqueous solution without soil (Laha et al., 2009). This higher dose of surfactant in the soil-water system is called elevated CMC or effective CMC (CMC_eff_). Above the CMC, a sharp increase in solubility is observed for that compound and the relationship between the solubility and concentration of surfactant above CMC is linear (Edwards et al., 1991). This indicates the excess amount of surfactant, the difference between the used amount of surfactant and the amount required to attain the CMC, creates increased micelle volume in the bulk solution that contributes to solubilizing higher concentrations of PAHs. Therefore, cleaning efficacy increases against all the PAH compounds when increased concentrations of CD-1 and CD-2 were used. The cleaning efficacy of both surfactants is very good against LMW PAHs when 1 mL of surfactant was applied. However, 1 mL of surfactant was not effective enough to solubilize HMW PAHs. Only 44% and 46% of HMW PAHs are removed by CD-1 and CD-2, respectively. Considering the low cleaning efficacy of 1 mL of surfactant, it can be assumed that 10 μL of CD-1 or CD-2 would not be able to solubilize or remove HMW PAHs from fabrics that are contaminated at the 60,000 ng level required by the NFPA 1851 standard for cleaning validation.

The cleaning efficacy of CD-1 and CD-2 increases sharply when 10 mL of surfactant is used. Almost all the LMW PAHs are removed with this concentration of both surfactants. Cleaning efficacies are around 84% and 75% against HMW PAHs for CD-1 and CD-2, respectively. Although 1 mL of CD-1 and CD-2 show similar cleaning efficacy, 10 mL of CD-1 shows better performance against HMW PAHs compared to CD-2. This indicates comparison among different surfactants is difficult as each surfactant has a different CMC value, contains different ingredients and is formulated differently by the manufacturer, which may or may not be optimized for a firefighter contamination application. Each surfactant requires a different concentration of surfactants to show optimum cleaning efficacy. Further increase of concentration (20 mL or 50 mL) of both surfactants does not show a sharp increase in cleaning efficacy against most of the HMW PAHs. Using 10 mL of surfactant was able to remove a high portion of the PAH compound. Therefore, cleaning efficacy did not increase sharply when 20 and 50 mL of surfactants were used. This also indicates the fabric’s surface is saturated with the micelle or bilayers with 10 mL of surfactants as the sorption of surfactants reaches the saturation point. As sorption reaches a plateau against most of the HMW PAHs, a nonlinear sorption isotherm is obtained against most of the HMW PAHs above 10 mL of surfactant in the fabric-surfactant-HOCs system (Zhou and Zhu, 2007). Although cleaning efficacy increased slowly above 10 mL of surfactants, even 50 mL of surfactant could not remove some of the HMW PAHs like Ind, D[ah]A, B[ghi]P ($K_{\text{OW}}$ value is above 6.5). PAHs with 6.5 or higher $K_{\text{OW}}$ value are strongly hydrophobic in nature, capable to be readily adsorbed on the fabric surface, and show a very high affinity to fabrics. If any surfactant can remove these three contaminants during washing, then it can be assumed that other PAH compounds will also be removed effectively. If the turnout gear is heavily contaminated with HMW PAHs, then firefighters may need to use a significantly high concentration of surfactants or a pre-soak step to effectively remove the contamination. The reported findings related to the cleaning of turnout gear from PAHs can be expected for other organic compounds like phenols and phthalates that are released during fires.

### 4.3 Cleaning efficacy of different cleaning products

In the aqueous solution, surfactants change the hydrophobicity of PAHs, which contributes to moving PAHs from fabric to hydrophobic micelle cores. The hydrophobicity of HOCs correlates with molecular weight and the number of aromatic rings present in the structure. Increasing the number of micelles in the aqueous solution will increase the washing performance. The previous section shows that hydrophobic factors, like $K_{\text{OW}}$ and solubility, play a significant role in removing PAHs from contaminated fabric. CD-2, CD-3, and CD-4 contain a blend of nonionic and anionic surfactants, and all three surfactants were found effective against PAHs when 10 mL of surfactant was used with 90 mL of water. Generally, synergism is observed when nonionic and anionic surfactants are mixed in the aqueous solution meaning that mixed micelles show improvement in some crucial properties including surface tension, solubility, and wettability compared to single micelles (Mohamed and Mahfoodh, 2006). Due to the synergism, mixed surfactants show lower surface/interfacial tensions, better micellar partition coefficients (K_m_), and require low concentration to reach CMC compared to single surfactants. The combined use of anionic and non-ionic surfactants reduces the polarity of micelles and increases the aggregation number (Shi et al., 2013). This contributes to higher solubilization of PAHs and more PAHs are transferred into the micelles of surfactant. The average cleaning efficacy of CD-2, CD-3, and CD-4 against ∑9 HMW PAHs are around 75%, 83%, and 83%, respectively. CD-1 contains nonionic surfactant and D-limonene. The repulsion force between the head groups of nonionic surfactants is relatively weak, enabling them to form large micelles in the aqueous solution. Therefore, nonionic surfactants showed better solubilization power to PAH compared to ionic surfactants (Liang et al., 2017). Besides that, the desorption of PAHs from the soil reached equilibrium at low CMC of nonionic surfactants compared to anionic surfactants. Therefore, the solubilization power of nonionic surfactant is also better compared to anionic surfactant (Shih et al., 2020). The cleaning performance of CD-1 is also boosted by the hydrophobic ingredient D-limonene to remove non-polar PAHs from fabrics. The average cleaning efficacy of CD-1 against ∑9 HMW PAHs is around 84%. All these surfactants removed more than 90% of ∑7 LMW PAHs.

CD-5, CD-6, CD-7, and CD-8 are charcoal-based shampoo or body wash. In this experiment, these products were used to decontaminate fabrics to investigate the cleaning efficacy of these products against PAHs. The high removal efficacy of PAHs from contaminated fabrics would indicate that these are capable of removing PAHs from body parts as well. Charcoal is a popular adsorbent due to its microporous structure, large specific surface area, higher surface activities, and high adsorption capacity (Gürses et al., 2016). One teaspoon of charcoal powder may have the same surface area as a football field (between 950 m^2^/g to 2000 m^2^/g) (Azmi et al., 2022). Therefore, charcoal acts like a magnet to grab contaminants from a contaminated surface. The average cleaning efficacy of CD-5, CD-6, CD-7, and CD-8 against ∑7 LMW PAHs is around 90% or higher. Above 70% cleaning efficacy is observed against ∑9 HMW PAHs for each of the cleaning products. All the cleaning products showed the lowest removal efficacy against D[ah]A due to having the highest $K_{\text{OW}}$ value among other PAHs. Results show that cleaning performance of charcoal-based products is similar to regular detergents in removing PAH compounds. The charcoal-based products also have a surfactant formulation in the structure, so it is hard to conclude whether the charcoal is removing PAHs from fabrics or it is the surfactants in the products that are removing the PAH compounds. Besides that, these charcoal-based products are developed to decontaminate the body parts of firefighters rather than fabric or turnout gear. Human skin can be divided into layers and each layer has different functions (Honari, 2017). PAHs that are generated at the fire scene can be adsorbed on the particles and deposited on the skin of firefighters. Human skin is prone to breakage and small particles or contaminants like PAHs can penetrate through the skin (Liyanage et al., 2021). Considering the complexities of skin, it is hard to predict the cleaning efficacy of charcoal-based products against PAHs deposited on the skin. Therefore, it is important to conduct further research to investigate whether the high removal efficacy of PAHs using charcoal-based cleaning products is also reflected when used to decontaminate the skin or body parts of firefighters.

## 5 Limitations

The laboratory-based washing method was used in this experiment as large numbers of samples needed to be washed separately. This bench-scale washing method may have some limitations including the sample size, and G-force applied during washing. The load size in the washing extractor during the laundry process is not comparable to the load size used in this laboratory-based washing method. Therefore, using the same ratio of detergents in washing extractors and laboratory-based washing methods may not provide the same results in cleaning performance. However, a similar trend should be observed in both washing processes. Girase et al. (2022) contaminated fabrics using some hydrophobic organic compounds (HOCs) including Py, and B[a]P. Then laundering of the contaminated gear was performed according to the current NFPA 1851 washing procedures using UNIMAC^®^ 45 lbs. washing extractor. The CD-1 was used during the washing process according to the manufacturer’s recommendation (120 mL CD-1 per 45 lbs load). The cleaning efficacies of these two HMW PAHs were 57.62% and 35.73%, respectively. This indicates cleaning efficacy against PAHs containing six aromatic rings would be much lower. Therefore, we can assume that performing laundry in a washing extractor and laboratory-based washing methods will show similar cleaning performance. This research showed that higher concentrations of detergent would be able to remove PAHs effectively. However, further research is required to investigate whether higher concentrations of surfactant would affect the functional or physical properties of turnout gear.

## 6 Conclusion

The molecular weight of PAHs is proportional to the solubility of PAHs and inversely proportional $K_{\text{OW}}$ values. PAHs with lower solubility and high $K_{\text{OW}}$ values show higher affinity to the fabric. Therefore, the removal efficacy of PAH compounds decreases with the increase of $K_{\text{OW}}$ values. HMW PAHs have higher $K_{\text{OW}}$ values compared to LMW PAHs. Therefore, HMW PAHs are difficult to remove compared to LMW PAHs. Water can remove a significant amount (around 61%) of LMW PAHs. However, detergents are critical to remove HMW PAHs from turnout gear. The observation indicates that surfactant-enhanced cleaning of PAH-contaminated gear largely depends on the concentrations of detergent during the washing process. The results indicate that the HMW-PAHs are the compounds that we need to focus on. The NFPA 1851 contains a list of contaminants in subsection 12.6. The PAHs in the list are LMW PAHs which are not much of a concern and do not represent the entire spectrum of PAHs. Thus, it is of crucial importance to modify the cleaning approach of the contaminants. This also includes in categorizing the compounds based on $K_{\text{ow}}$ values which will help in developing more targeted approach towards verifying the independent service providers (ISP)s. Using detergents based on the load size may not remove PAHs from turnout gear effectively if the gear is highly contaminated with organic compounds like PAHs. This research will help firefighters and fire services understand that they may need to use a higher concentration of surfactant than the recommended quantity by detergent manufacturers to effectively remove PAHs from turnout gear. Although the study highlighted that the concentration of the surfactants plays a vital role in removing PAHs, the effect of these concentrations on the PPE need to be studied. Since the high $K_{\text{ow}}$ values indicate the high affinity of the compounds towards the organic matter than the water then these compounds will partition more towards particulate matter. At such times, the on-site decontamination technique if used with high concentration of surfactant and brushing of the particulate matter that can help in achieving the maximum decontamination of HMW-PAHs. Evaluation of the cleaning performance of regular detergents showed that using only nonionic or the combined use of nonionic and anionic surfactants can remove PAHs from turnout gear effectively. Charcoal-based cleaning products are also found effective to remove PAHs from turnout gear. Therefore, it can be assumed that using charcoal-based products might be a useful technique to remove PAHs from the body parts of firefighters if the skin of firefighters is contaminated by PAHs.

## Supplementary Material

Table 1

Image 1 **SUPPLEMENTARY FIGURE S1** Cleaning efficacy of regular detergents against LMW PAHs [range=mean ± SE].

Image 2 **SUPPLEMENTARY FIGURE S2** Cleaning efficacy of charcoal-based detergents against LMW PAHs [range=mean ± SE].

Table 2

## Figures and Tables

**TABLE 1:** Chemical and physical properties of 16 targeted PAHs (ATSDR, 2005; Bojes and Pope, 2007; Patel et al., 2020).

PAH compound	Molecular weight (g/mol)	Number of benzene rings	Boiling point (°C)	Octanol-water partitioning coefficient log K_ow_	Solubility in water (mg/L)	LOD (ng/μL)	LOQ (ng/μL)
Naphthalene (Nap)	128.17	2	218	3.29	31	0.10	0.33
Acenaphthylene (Acy)	152.2	3	280	4.07	3.8	0.03	0.10
2-Bromo naphthalene (2-Br)	207	2	281	No data	3.4	0.03	0.09
Acenaphthene (Ace)	154.21	3	279	3.98	0.045	0.03	0.12
Fluorene (Fle)	166.22	3	295	4.18	1.9	0.03	0.11
Phenanthrene (PHE)	178.23	3	340	4.45	1.1	0.04	0.12
Anthracene (An)	178.23	3	340	4.45	0.045	0.03	0.10
Fluoranthene (Fla)	202.25	4	404	4.9	0.26	0.03	0.09
Pyrene (Py)	202.26	4	400	4.88	0.132	0.02	0.07
Benz a anthracene B [a]A	228.29	4	438	5.61	0.011	0.03	0.11
Chrysene (Chr)	228.29	4	448	5.9	0.0015	0.02	0.05
Benzo b fluoranthene B[b]F	252.32	5	481	6.04	0.0015	0.03	0.09
Benzo a pyrene B[a]P	252.32	5	495	6.06	0.0038	0.03	0.09
Indeno 1,2,3-cd pyrene (Ind)	276.33	6	530	6.58	0.062	0.03	0.10
Dibenz a,h anthracene D[ah]A	278.35	6	524	6.84	0.0005	0.06	0.19
Benzo g,h,i perylene B[ghi]P	276.33	6	550	6.5	0.00026	0.07	0.22

**TABLE 2:** Calibration standard preparation for chromatography method.

Calibration standard	Target concentration (ng/μL)	The volume injected from the stock solution (μL)	Mass per unit fabric area (ng/cm^2^)
1	0.2	1	100
2	0.8	4	400
3	2	10	1000
4	4	20	2000
5	6	30	3000
6	8	40	4000

## Data Availability

The original contributions presented in the study are included in the article/Supplementary Material, further inquiries can be directed to the corresponding authors.
