# Supplementary material for: Evaluating the performance of surfactant and charcoal-based cleaning products to effectively remove PAHs from firefighter gear
Source: Front Mater. Author manuscript; Available in PMC 2023 Dec 6. (PMC10698686; doi:10.3389/fmats.2023.1142777)
Supplement: Table 1 [file NIHMS1925740-supplement-Table_1.doc]

**Supplementary Table 1**: List of cleaning products with their concentrations used in the experiment

| **Detergents** | **Concentration of surfactant used in experiments (mL)** | | | | |
| --- | --- | --- | --- | --- | --- |
| CD-1 | 0* | 1 | 10 | 20 | 50 |
| CD-2 | 0* | 1 | 10 | 20 | 50 |
| CD-3 | - | - | 10 | - | - |
| CD-4 | - | - | 10 | - | - |
| CD-5 | - | - | 10 | - | - |
| CD-6 | - | - | 10 | - | - |
| CD-7 | - | - | 10 | - | - |
| CD-8 | - | - | 10 | - | - |

0*= water-only experiments
