# Supplementary figures and images for: Evaluating the performance of surfactant and charcoal-based cleaning products to effectively remove PAHs from firefighter gear
Source: Front Mater. Author manuscript; Available in PMC 2023 Dec 6. (PMC10698686; doi:10.3389/fmats.2023.1142777)

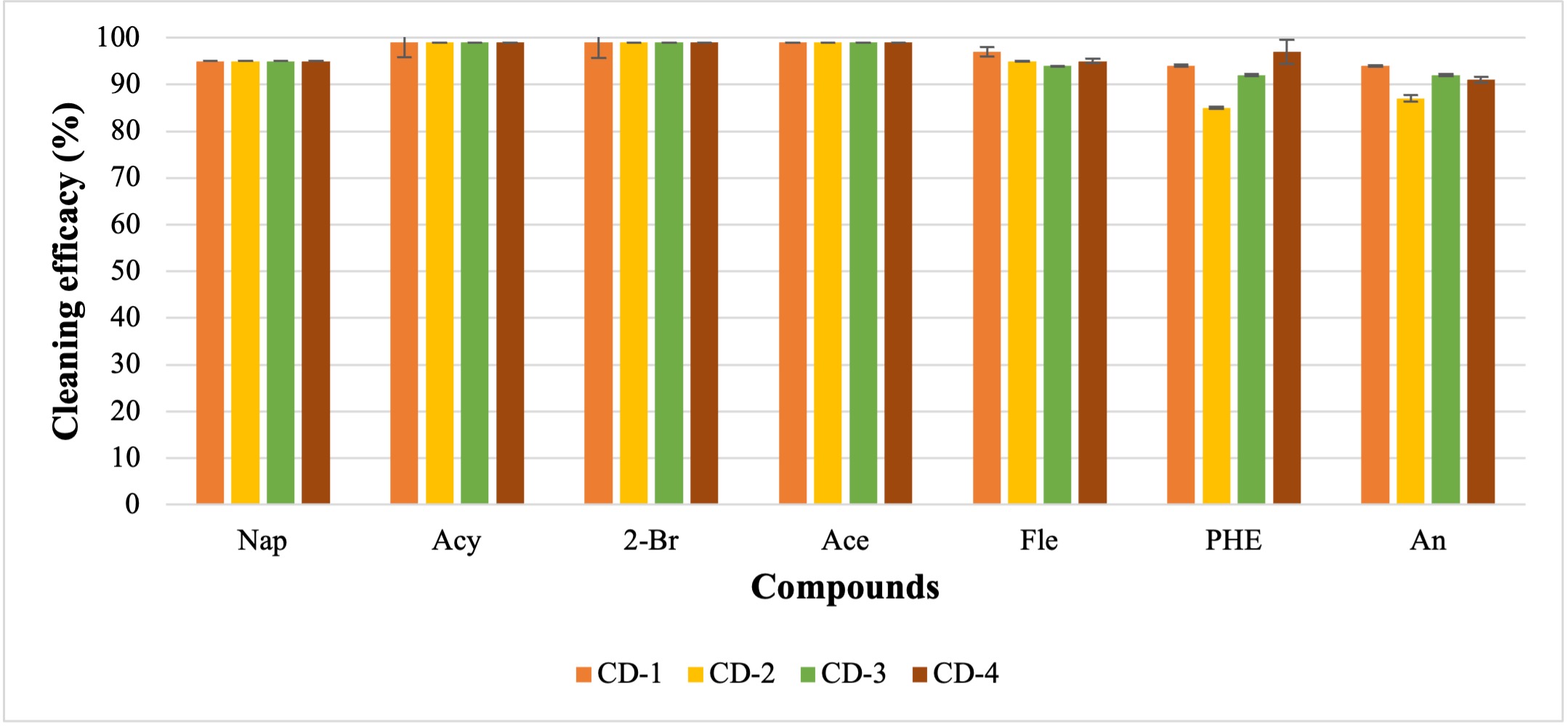

Supplement: Image 1 — SUPPLEMENTARY FIGURE S1 Cleaning efficacy of regular detergents against LMW PAHs [range=mean ± SE]. [file NIHMS1925740-supplement-Image_1.jpg]

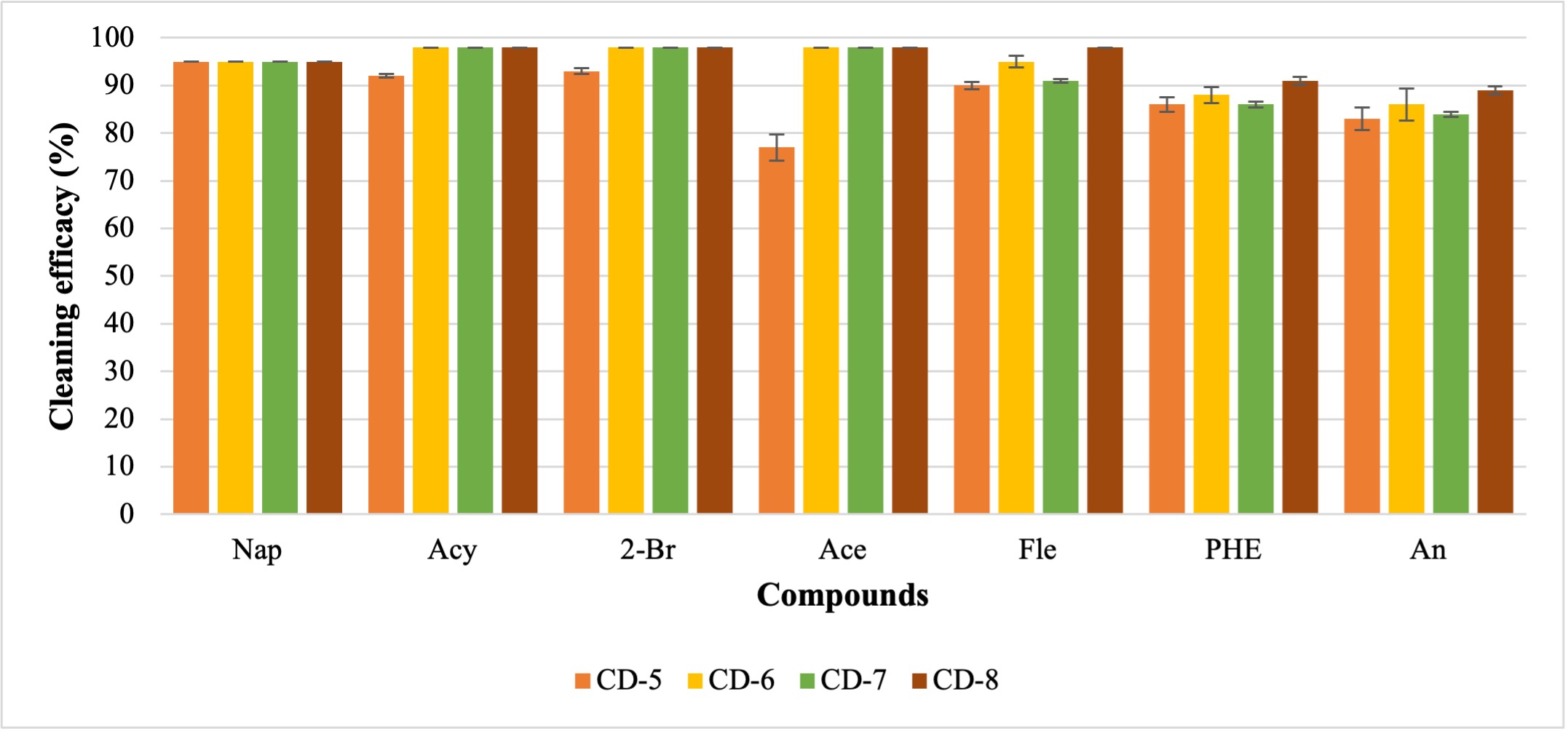

Supplement: Image 2 — SUPPLEMENTARY FIGURE S2 Cleaning efficacy of charcoal-based detergents against LMW PAHs [range=mean ± SE]. [file NIHMS1925740-supplement-Image_2.jpg]
