# Supplementary material for: Evaluating the performance of surfactant and charcoal-based cleaning products to effectively remove PAHs from firefighter gear
Source: Front Mater. Author manuscript; Available in PMC 2023 Dec 6. (PMC10698686; doi:10.3389/fmats.2023.1142777)
Supplement: Table 2 [file NIHMS1925740-supplement-Table_2.doc]

**Supplementary Table 2:** Cleaning efficacy of CD-2 against 16 PAHs

|  | **Average cleaning efficacy (%) of different concentrations of CD-2** | | | | | | | | |
| --- | --- | --- | --- | --- | --- | --- | --- | --- | --- |
|  | **1 mL** | **Avg SE for 1 mL** | **10 mL** | **Avg SE for 10 mL** | **20 mL** | **Avg SE for 20 mL** | **50 mL** | **Avg SE for 50 mL** |  |
| Nap | 95 | 0 | 95 | 0 | 95 | 0 | 99 | 0 |  |
| Acy | 86 | 0.72 | 99 | 0 | 99 | 0 | 99 | 0 |  |
| 2-Br | 83 | 0.69 | 99 | 0 | 99 | 0 | 99 | 0 |  |
| Ace | 89 | 0.16 | 99 | 0 | 99 | 0 | 99 | 0 |  |
| Fle | 78 | 1.79 | 95 | 0.09 | 96 | 1 | 95 | 0.47 |  |
| PHE | 65 | 3.12 | 85 | 0.17 | 91 | 2.5 | 91 | 0.06 |  |
| An | 61 | 2.94 | 87 | 0.65 | 90 | 2.82 | 90 | 0.45 |  |
| Fla | 61 | 3.77 | 87 | 0.16 | 92 | 1.48 | 92 | 0.2 |  |
| Py | 62 | 4.06 | 88 | 0.05 | 92 | 1.35 | 92 | 0.18 |  |
| B[a]A | 50 | 3.63 | 80 | 0.1 | 86 | 3.25 | 86 | 0.85 |  |
| Chr | 38 | 3.59 | 69 | 0.89 | 77 | 6.93 | 75 | 1.64 |  |
| B[b]F | 46 | 3.64 | 76 | 0.37 | 83 | 3.32 | 85 | 0.63 |  |
| B[a]P | 47 | 3.59 | 77 | 0.35 | 84 | 2.44 | 85 | 0.67 |  |
| Ind | 41 | 3.81 | 70 | 0.4 | 82 | 2.57 | 84 | 0.61 |  |
| D[ah]A | 27 | 3.29 | 53 | 0.93 | 64 | 5.53 | 69 | 1.47 |  |
| B[ghi]P | 41 | 3.72 | 73 | 0.47 | 80 | 2.77 | 82 | 0.73 |  |
